# Genetic Variability in L1 and L2 Genes of HPV-16 and HPV-58 in Southwest China

**DOI:** 10.1371/journal.pone.0055204

**Published:** 2013-01-25

**Authors:** Yaofei Yue, Hongying Yang, Kun Wu, Lijuan Yang, Junying Chen, Xinwei Huang, Yue Pan, Youqing Ruan, Yujiao Zhao, Xinan Shi, Qiangming Sun, Qihan Li

**Affiliations:** 1 Institute of Medical Biology, Chinese Academy of Medical Sciences, and Peking Union Medical College, Kunming, People's Republic of China; 2 Yunnan Key Laboratory of Vaccine Research and Development on Severe Infectious Diseases, Kunming, People's Republic of China; 3 The Third Affiliated Hospital of Kunming Medical University (Yunnan Provincial Tumor Hospital), Kunming, People's Republic of China; 4 The First Affiliated Hospital of Kunming Medical University, Kunming, People's Republic of China; 5 Southwest Guizhou Vocational and Technical College for Nationalities, Xingyi, People's Republic of China; IPO, Inst Port Oncology, Portugal

## Abstract

HPV account for most of the incidence of cervical cancer. Approximately 90% of anal cancers and a smaller subset (<50%) of other cancers (oropharyngeal, penile, vaginal, vulvar) are also attributed to HPV. The L1 protein comprising HPV vaccine formulations elicits high-titre neutralizing antibodies and confers type restricted protection. The L2 protein is a promising candidate for a broadly protective HPV vaccine. In our previous study, we found the most prevalent high-risk HPV infectious serotypes were HPV-16 and HPV-58 among women of Southwest China. To explore gene polymorphisms and intratypic variations of HPV-16 and HPV-58 L1/L2 genes originating in Southwest China, HPV-16 (L1: n = 31, L2: n = 28) and HPV-58 (L1: n = 21, L2: n = 21) L1/L2 genes were sequenced and compared to others described and submitted to GenBank. Phylogenetic trees were then constructed by Neighbor-Joining and the Kimura 2-parameters methods (MEGA software), followed by an analysis of the diversity of secondary structure. Then selection pressures acting on the L1/L2 genes were estimated by PAML software. Twenty-nine single nucleotide changes were observed in HPV-16 L1 sequences with 16/29 non-synonymous mutations and 13/29 synonymous mutations (six in alpha helix and two in beta turns). Seventeen single nucleotide changes were observed in HPV-16 L2 sequences with 8/17 non-synonymous mutations (one in beta turn) and 9/17 synonymous mutations. Twenty-four single nucleotide changes were observed in HPV-58 L1 sequences with 10/24 non-synonymous mutations and 14/24 synonymous mutations (eight in alpha helix and four in beta turn). Seven single nucleotide changes were observed in HPV-58 L2 sequences with 4/7 non-synonymous mutations and 3/7 synonymous mutations. The result of selective pressure analysis showed that most of these mutations were of positive selection. This study may help understand the intrinsic geographical relatedness and biological differences of HPV-16/HPV-58 and contributes further to research on their infectivity, pathogenicity, and vaccine strategy.

## Introduction

Human Papillomavirus (HPV) virions are one of the most important pathogenic agents for cervical cancer, which accounts for a worldwide cancer burden in women second only to breast cancer [1], [2]. Approximately 90% of anal cancers and a smaller subset (<50%) of other cancers (oropharyngeal, penile, vaginal, and vulvar) are also attributed to HPV. In total, HPV accounts for 5.2% of the worldwide cancer burden. HPVs 16 and 18 are responsible for 70% of cervical cancer cases and, especially HPV-16, for a large proportion of other cancers [3]. On the basis of their oncogenic potential, HPV types that infect the genital tract are classified as low risk (LR) and high risk (HR) [4]. Low-risk HPVs (including HPV-6, 11, 42, 43, and 44) are mainly associated with benign genital warts, while high-risk HPVs (including HPV-16, 18, 31, 33, 39, 45, 51, 52, 56, 58, 59, and 68) are the etiological agents of cervical cancer, a disease that affects approximately 500,000 women worldwide [5]. In our previous study, we found the most prevalent high-risk HPV infectious serotypes were HPV-16 and HPV-58 among women of Southwest China.

The HPV genome is packaged within a non-enveloped, icosahedral capsid composed of 72 pentamers of the major capsid late protein (L1) and an unknown number of the minor capsid proteins L2 [6], [7]. The pentamers of L1 expressed in heterologous systems that assemble into virus-like particles (VLPs) [8] are the components used in the design of prophylactic vaccines. The L1 protein comprising HPV vaccine formulations elicits high-titre neutralizing antibodies and confers type-specific and long-lasting protection against persistent infection and associated cervical neoplasia attributable to HPV vaccine types [8], [9]. However, there has been no vaccine designed that can prevent all HPV infections owing to the lack of cross-reactivity between L1 proteins of different HPV types.

The inner conical hollow of L1 pentamers can be occluded with a monomer of L2 [6]. An N-terminal “external loop” of L2 contains cross-neutralizing epitopes, which can be the target of neutralizing and cross-neutralizing antibodies as well [10]–[12]. Analysis of FUTURE I/II and PATRICIA data suggested cross-protective vaccine efficacy against infections and lesions associated with HPV 31, 33, and 45 [13]. Therefore, targeting L2 may be an acceptable approach for a candidate vaccine. However, its low abundance in natural capsids (only 12–72 molecules per 360 copies of L1) limits its immunogenicity [14]. Currently, we have no efficient vaccines against L2 to prevent infection with these high-risk HPV types.

Due to the high prevalence of HPV not only among asymptomatic women but also in samples of different neoplasias worldwide, the association between intratypical variants of HPV-16 L1 has been described in several papers. Nevertheless, data concerning molecular variants of HPV-16 and HPV-58 L2 are still limited, necessitating further studies that would be essential to expand knowledge of the different variants. Furthermore, there is little data regarding the intratypical variants of HPV-16 L1 in Southwest China.

The aim of this study is to detect the nucleotide variability, gene polymorphism and phylogeny in the L1 and L2 genes of the High-Risk HPV-16 (L1: n = 31, L2: n = 28) and HPV-58 (L1: n = 21, L2: n = 21) samples obtained from Southwest China. The most variable sequences were chosen for an analysis of the diversity of their secondary structure. Nucleotide and amino acid sequence alignments were used to evaluate variant clusters. Amino acid changes of L1 and L2 genes might affect immune responses to HPV-16 and HPV-58 capsid proteins and advance HPV vaccine strategies. The genomic characterization of HPV variants is pivotal for a deeper understanding of the intrinsic geographical relatedness and biological differences of these viruses and contributes further to research on their infectivity and pathogenicity.

## Materials and Methods

### Ethics Statement

Written consent was obtained from each participant. The study protocol was approved by the institutional ethics committee (Institute of Medical Biology, Chinese Academy of Medical Sciences, and Peking Union Medical College) and was in accordance with the Declaration of Helsinki for Human Research of 1974 (last modified in 2000).

### Clinical Specimens

Samples examined in this study were obtained from cervical scrapings of 3000 volunteer outpatients from women in Southwest China from 2009 to 2011. After routine cytology and HC2 testing, a cell suspension from each sample was placed in a 1.5 mL Eppendorf tube and transferred to a laboratory at the Institute of Medical Biology for HPV DNA amplification. HPV typing of these specimens was performed using a nested multiplex PCR assay as described previously [15], [16]. HPV-16 (L1: n = 31; L2: n = 28), HPV-58 (L1: n = 21; L2: n = 21) sequences thereby obtained were used for molecular characterization by sequence analysis of the L1 and L2 gene.

### Nucleic acid extraction and sequencing amplification

Molecular characterization was performed by sequence analysis of L1 and L2 gene amplicons. The entire region of L1 and L2 genes of HPV-16 and HPV-58 were amplified using degenerate primer pairs. Two partially overlapping fragments for each virus were amplified. All Primers were designed and synthesized by Sangon Biotech (Shanghai). The GeneBank reference sequences used for primer design were HPV-16 (NC001526 [17]) and HPV-58 (HQ537759). The primer sequences and their relative positions are listed in Table 1. The amplification of the fragments was performed in 50 ul reaction volumes containing 5 ul extracted DNA (template), 2×power taq PCR MasterMix (TaKaRa), 25 pmol of each primer (Sangon Biotech) and deionized water. The cycling conditions were as follows: 94° for 5 min followed by 30 cycles at 94° for 45 sec, 50° for 45 sec, 72° for 1 min and a final 72° extension for 7 min. Amplicons were visualized on 2% agarose gels stained with GoldViewTM Nucleic Acid Stain. Then PCR products were purified and sequenced by Sangon Biotech.

**Table 1:** Primers used for the molecular characterization of HPV-16 and HPV-58 L1 and L2.

Primer name	Sequence 5′ to 3′	Amplicon size (bp)
HPV16 L1 1F	ATAGTTCCAGGGTCTCCA	743
HPV16 L1 1R	AGTCCATAGCACCAAAGC	
HPV16 L1 2F	GAACACTGGGGCAAAGGATC	1236
HPV16 L1 2R	TACAATGAATAACCACAACA	
HPV16 L2 1F	TTACTTAACAATGCGACACA	801
HPV16 L2 1R	TTATCCACATCTATACCTTCA	
HPV16 L2 2F	CCCTGCTTTTGTAACCACTC	776
HPV16 L2 2R	CGTGCAACATATTCATCCGT	
HPV58 L1 1F	GTGTCATTGGAACCTGGTCCA	985
HPV58 L1 1R	GCCAAGTTTTCCAGCCCTATT	
HPV58 L1 2F	GCCAGTGAACCTTATGGGGAT	771
HPV58 L1 2R	TTTGCGTTTGGTGGATGGT	
HPV58 L2 1F	ATGGTGGTATGGTATTGT	810
HPV58 L2 1R	CTTAACTTGTTGGGTGGT	
HPV58 L2 2F	TCCTTTACTGAGCCATCC	1018
HPV58 L2 2R	ATAAATGCTTGTGCGTGA	

### Molecular characterization and phylogenetic analysis

All sequences from a given sample were combined and used to construct alignments. ClustalX (version 1.83) multiple sequence alignment analysis was carried out to calculate the percentage of sequence similarity between the L1/L2 amplicons and the representative sequences of the HPV-16 and HPV-58 variants. Phylogenetic trees of respective HPV-16 and HPV-58 L1 and L2 sequences were constructed by the Neighbor-Joining method [18]–[20] and the Kimura 2-Parameter model by MEGA package, version4.1. A bootstrap re-sampling analysis was performed (2,000 replicates) to test the robustness of the major phylogenetic groups [17], [19], [21]. To estimate selection pressure acting on the HPV-16 and HPV-58 L1 and L2 gene sequences, synonymous and nonsynonymous nucleotide divergence for coding regions was inferred by the method of Nei and Gojobori [21]–[23] with PAML 4.0. The L1 and L2 gene sequences of the HPV-16, and 58 viral strains studied were deposited into the NCBI GenBank database (http://www.ncbi.nim.nih.gov/GenBank/index.html): JX313693-JX313793. The reference viral sequences that were used to construct the distinct phylogenetic branches were collected from the GenBank sequence database under the following accession numbers: HPV-16: AY686580, FJ006723 (China, Xinjiang), AY686581, EU118173, AF534061, U89348, AF536179, AF472508, AF536180, AF472509, and AY686579 [16]; HPV-58: D90400 (Japan), EU918765, HQ537760 (isolate AS347), HQ537762, HQ537763, HQ537776, HQ537774, and HQ537777.

## Results

### Phylogenetic and amino acid mutations analysis of HPV-16 L1 sequences

**Figure 1:**
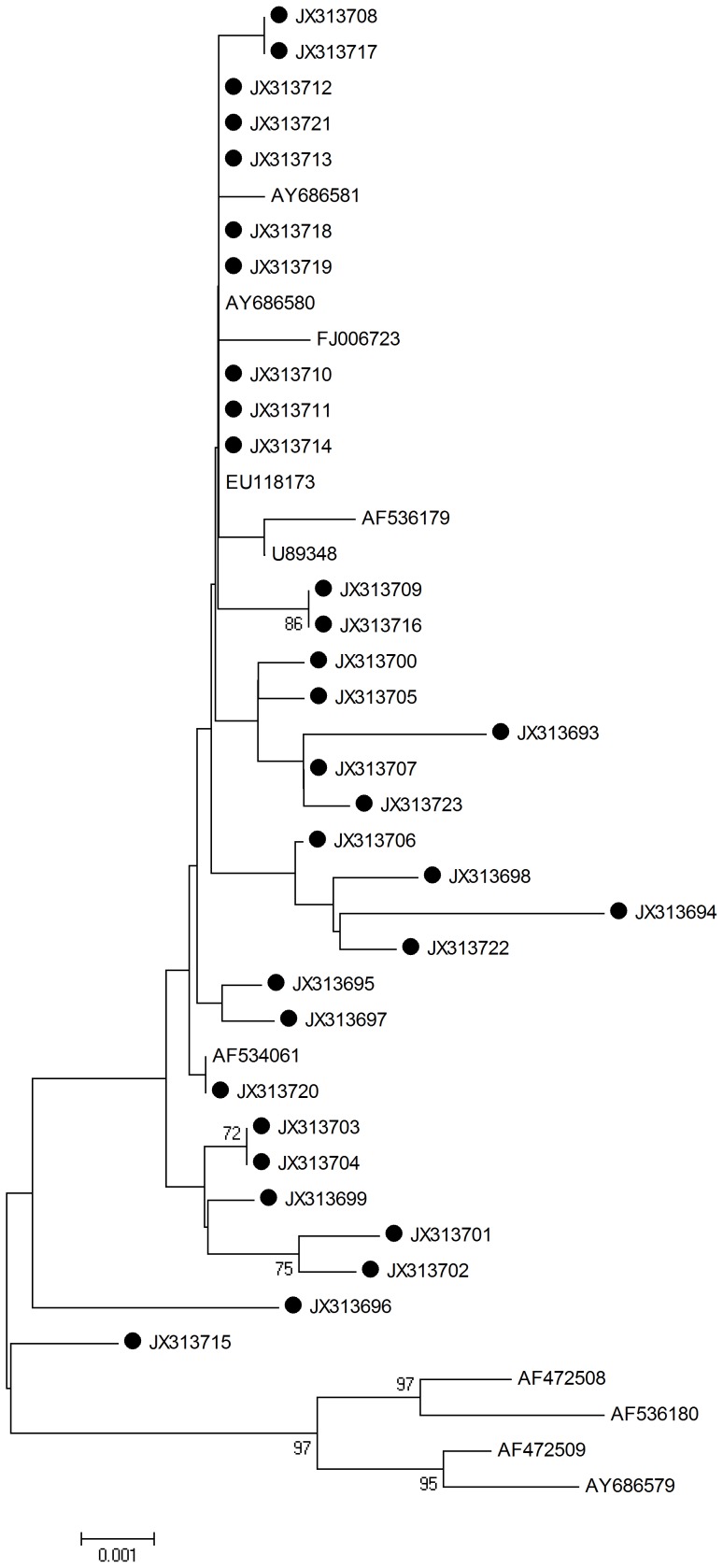
Neighbor joining phylogenetic tree generated using the nucleotide sequences of HPV-16 L1 gene. Study sequences are labeled in black. Others are standard sequences, including: AY686580, FJ006723 (China, Xinjiang), AY686581, EU118173, AF534061, U89348, AF536179, AF472508, AF536180, AF472509, and AY686579. Phylogenetic trees were constructed by the Neighbor-Joining method and the Kimura 2-Parameter model by MEGA package.

L1 HPV-16 sequences were determined and analyzed by aligning L1 1596 nucleotide sequences from all viral strains (n = 31; including the reference sequences). The neighbor joining phylogenetic tree can be seen in Fig. 1.

Twenty-nine single nucleotide changes were identified among the sequences studied. Specifcally, 13/29 (44.8%) were synonymous mutations and 16/29 (55.2%) were non-synonymous mutations. Of the 6 amino acid mutations observed in the sequences encoding the alpha helix, only one was a non-synonymous mutation. 2 non-synonymous mutations were observed in the sequences encoding the beta turn (glycine to arginine) (http://npsa-pbil.ibcp.fr/cgi-bin/npsa_automat.). 1 of 31 sequences did not belong to any standard type branch (Fig. 1). 7 samples were found to have the same mutation from A to C at the position of 979. The detected mutations are summarized in Table 2.

**Table 2:** Nucleotide sequence mutations of HPV-16 L1.

	Domain: HPV-16 L1 sequence																											
																					1	1	1	1	1	1	1	1
		1	1	1	1	1	1	2	3	3	4	7	9	9	9	9	9	9	9	9	1	2	3	4	4	5	5	5
	5	0	0	0	0	6	6	9	6	9	2	1	4	7	7	7	8	8	8	9	3	6	2	7	9	0	2	6
	2	2	3	4	7	5	8	9	6	9	3	4	5	7	8	9	1	2	3	0	2	6	9	8	5	7	5	6
NC001526	G	G	G	C	C	A	C	G	G	A	G	A	A	T	T	A	C	T	C	C	G	C	T	A	G	A	G	A
JX313693	.	C	T	A	A	.	.	.	.	.	.	.	.	.	.	.	.	.	.	.	.	T	G	.	.	.	.	.
JX313694	A	.	T	A	.	.	.	T	A	.	.	.	.	.	.	.	.	.	.	.	A	.	.	.	.	.	C	C
JX313695	.	.	.	.	.	.	.	.	.	.	.	.	.	.	.	.	.	.	.	.	.	.	.	G	.	.	.	.
JX313696	.	.	.	.	.	.	.	.	.	.	A	.	.	C	C	C	.	.	.	.	.	T	.	.	.	.	.	.
JX313697	.	.	.	.	.	.	.	.	.	.	.	.	G	.	.	.	.	.	.	.	.	.	.	G	.	.	.	.
JX313698	.	.	.	.	.	.	.	.	.	.	.	.	.	.	A	C	.	.	.	.	A	.	.	.	.	.	C	C
JX313699	.	.	.	.	.	.	.	.	.	.	.	.	.	.	A	C	.	.	.	.	.	.	.	G	.	.	.	.
JX313700	.	.	.	.	.	.	A	.	.	.	.	.	.	.	.	.	.	.	.	.	.	T	.	.	.	.	.	.
JX313701	.	.	.	.	.	.	.	.	.	.	.	.	.	.	A	C	T	C	.	.	.	T	G	.	.	.	.	.
JX313702	.	.	.	.	.	.	.	.	.	.	.	.	.	.	A	C	T	C	T	.	.	.	.	.	.	.	.	.
JX313703	.	.	.	.	.	.	.	.	.	.	.	.	.	.	A	C	.	.	.	.	.	.	.	.	.	.	.	.
JX313704	.	.	.	.	.	.	.	.	.	.	.	.	.	.	A	C	.	.	.	.	.	.	.	.	.	.	.	.
JX313705	.	.	.	.	.	.	.	.	.	.	.	.	.	.	.	.	.	.	.	A	.	T	.	.	.	.	.	.
JX313706	.	.	.	.	.	.	.	.	.	.	.	.	.	.	.	.	.	.	.	.	A	.	.	.	.	.	.	C
JX313707	.	.	.	.	.	.	.	.	.	.	.	.	.	.	.	.	.	.	.	.	.	T	G	.	.	.	.	.
JX313708	.	.	.	.	.	C	.	.	.	.	.	.	.	.	.	.	.	.	.	.	.	.	.	.	.	.	.	.
JX313709	.	.	.	.	.	.	.	.	.	.	.	G	.	.	.	.	.	.	.	.	.	.	.	.	.	C	.	.
JX313710	.	.	.	.	.	.	.	.	.	.	.	.	.	.	.	.	.	.	.	.	.	.	.	.	.	.	.	.
JX313711	.	.	.	.	.	.	.	.	.	.	.	.	.	.	.	.	.	.	.	.	.	.	.	.	.	.	.	.
JX313712	.	.	.	.	.	.	.	.	.	.	.	.	.	.	.	.	.	.	.	.	.	.	.	.	.	.	.	.
JX313713	.	.	.	.	.	.	.	.	.	.	.	.	.	.	.	.	.	.	.	.	.	.	.	.	.	.	.	.
JX313714	.	.	.	.	.	.	.	.	.	.	.	.	.	.	.	.	.	.	.	.	.	.	.	.	.	.	.	.
JX313715	.	.	.	.	.	.	.	.	.	.	.	G	.	.	.	.	.	.	.	.	.	.	.	.	.	C	.	.
JX313716	.	.	.	.	.	.	.	.	.	.	.	G	.	.	.	.	.	.	.	.	.	.	.	.	.	C	.	.
JX313717	.	.	.	.	.	C	.	.	.	.	.	.	.	.	.	.	.	.	.	.	.	.	.	.	.	.	.	.
JX313718	.	.	.	.	.	.	.	.	.	.	.	.	.	.	.	.	.	.	.	.	.	.	.	.	.	.	.	.
JX313719	.	.	.	.	.	.	.	.	.	.	.	.	.	.	.	.	.	.	.	.	.	.	.	.	.	.	.	.
JX313720	.	.	.	.	.	.	.	.	.	.	.	.	.	.	.	.	.	.	.	.	.	.	.	.	.	.	.	.
JX313721	.	.	.	.	.	.	.	.	.	.	.	.	.	.	.	.	.	.	.	.	.	.	.	.	.	.	.	.
JX313722	.	.	.	.	.	.	.	.	.	C	.	.	.	.	.	.	.	.	.	.	A	.	.	.	.	.	C	C
JX313723	.	.	.	.	.	.	.	.	.	.	.	.	.	.	.	.	.	.	.	.	.	T	G	.	C	.	.	.
AA mutation	.	E-D	A-Y(35)	A-Y(35)	T-N			R-L						V-A		T-P(327)	T-P(327)	S-P	S-L		E-K				G-R	K-Q	G-R	
second structure	Alpha helix					Alpha helix	Alpha helix															Alpha helix	Alpha helix	Alpha helix	Beta turn		Beta turn	

Compared to prototype HPV sequences, insertion and deletion events were not identified and there was no evidence of premature stop codons or nucleotide deletions in the L1 HPV-16 sequences analyzed.

### Phylogenetic and amino acid mutations analysis of HPV-16 L2 sequences

**Figure 2:**
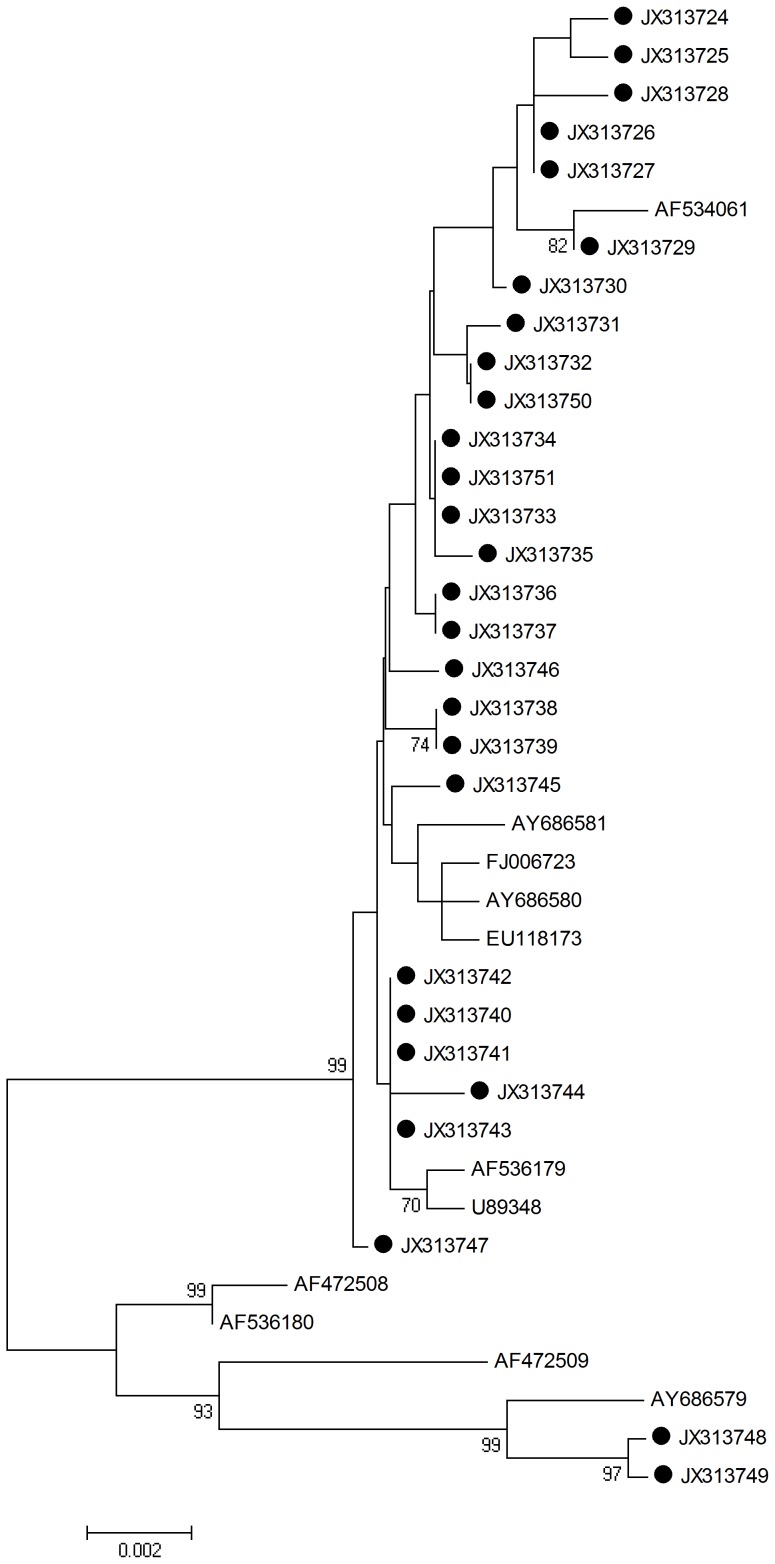
Neighbor joining phylogenetic tree generated using nucleotide sequences of the HPV-16 L2 gene. Study sequences are labeled in black. Others are standard sequences, including: AY686580, FJ006723 (China, Xinjiang), AY686581, EU118173, AF534061, U89348, AF536179, AF472508, AF536180, AF472509, and AY686579. Phylogenetic trees were constructed by the Neighbor-Joining method and the Kimura 2-Parameter model by MEGA package.

L2 HPV-16 sequences were determined and analyzed by aligning L2 1422 nucleotide sequences from all viral strains (n = 28; including the reference sequences). The neighbor joining phylogenetic tree can be seen in Fig. 2.

Seventeen single nucleotide changes were identified among the sequences studied. Specially, 9/17 (52.9%) were synonymous mutations and 8/17 (47.1%) were non-synonymous mutations. No amino acid changes were discovered at residues 65–71 and 112–120, which play a important role in inducing neutralizing antibodies [24]. No amino acid mutations occurred in the sequences encoding the alpha helix. Only one non-synonymous mutation was observed in the sequences encoding the beta turn (aspartic acid to glutamic acid). Both JX313748 and JX313749 L2 sequences fell into the same branch of AY686579 (Fig. 2). The detected mutations are summarized in Table 3.

**Table 3:** Nucleotide sequence mutations of HPV-16 L2.

	Domain: HPV-16 L2 sequence																
											1	1	1	1	1	1	1
	1	3	7	7	7	7	8	9	9	9	0	0	0	1	2	2	3
	9	6	4	7	8	8	7	1	5	9	1	5	7	1	3	3	9
	2	3	7	4	1	3	6	6	4	5	8	9	4	3	0	9	6
NC001526	G	A	T	A	G	T	A	A	A	C	G	A	T	T	A	T	T
JX313724	.	.	.	.	A	.	.	.	.	.	C	.	.	.	.	.	.
JX313725	.	.	C	.	A	.	.	.	.	.	.	.	.	.	.	.	.
JX313726	.	.	.	.	.	.	.	.	.	.	.	.	.	.	.	.	.
JX313727	.	.	.	.	.	.	.	.	.	.	.	.	.	.	.	.	.
JX313728	.	.	.	T	.	.	T	.	.	.	.	.	.	.	.	.	.
JX313729	.	.	.	.	.	.	.	.	.	.	.	.	.	.	.	.	.
JX313730	.	.	.	.	.	.	.	.	.	.	.	.	.	.	.	.	.
JX313731	.	.	.	.	.	.	.	.	.	.	.	.	.	.	G	.	.
JX313732	.	.	.	.	.	.	.	.	.	.	.	.	.	.	G	.	.
JX313733	.	.	.	.	.	.	.	.	.	.	.	.	.	.	.	.	.
JX313734	.	.	.	.	.	.	.	.	.	.	.	.	.	.	.	.	.
JX313735	.	.	.	.	.	G	.	.	.	.	.	.	.	.	.	.	.
JX313736	.	.	.	.	.	.	.	.	.	.	.	.	.	.	.	.	.
JX313737	.	.	.	.	.	.	.	.	.	.	.	.	.	.	.	.	.
JX313738	.	.	.	.	.	.	.	.	.	A	.	.	.	.	.	.	.
JX313739	.	.	.	.	.	.	.	.	.	A	.	.	.	.	.	.	.
JX313740	.	.	.	.	.	.	.	.	.	.	.	.	.	.	.	.	.
JX313741	.	.	.	.	.	.	.	.	.	.	.	.	.	.	.	.	.
JX313742	.	.	.	.	.	.	.	.	.	.	.	.	.	.	.	.	.
JX313743	.	.	.	.	.	.	.	.	.	.	.	.	.	.	.	.	.
JX313744	.	.	.	.	.	.	.	.	C	.	.	.	.	.	.	.	C
JX313745	.	.	.	.	.	.	.	C	.	.	.	.	.	.	.	.	.
JX313746	.	.	.	.	.	.	.	.	.	.	.	.	.	G	.	.	.
JX313747	.	.	.	.	.	.	.	.	.	.	.	.	.	.	.	.	.
JX313748	T	C	.	.	.	.	.	.	.	.	.	C	A	.	.	A	.
JX313749	C	C	.	.	.	.	.	.	.	.	.	C	A	.	.	A	.
JX313750	.	.	.	.	.	.	.	.	.	.	.	.	.	.	G	.	.
JX313751	.	.	.	.	.	.	.	.	.	.	.	.	.	.	.	.	.
AA mutation				E-D	D-N	D-E			K-N	T-N	E-Q			D-E			F-L
second structure	Random coil	Random coil	Extended strand.	Random coil	Extended strand.	Extended strand.	Random coil	Random coil	Random coil	Random coil	Extended strand.	Random coil	Random coil	Beta turn	Extended strand.	Random coil	Extended strand.

Insertion and deletion events were not present and there was no evidence of premature stop codons or nucleotide deletions within the L2 HPV-16 analyzed sequences.

### Phylogenetic and amino acid mutations analysis of HPV-58 L1 sequences

**Figure 3:**
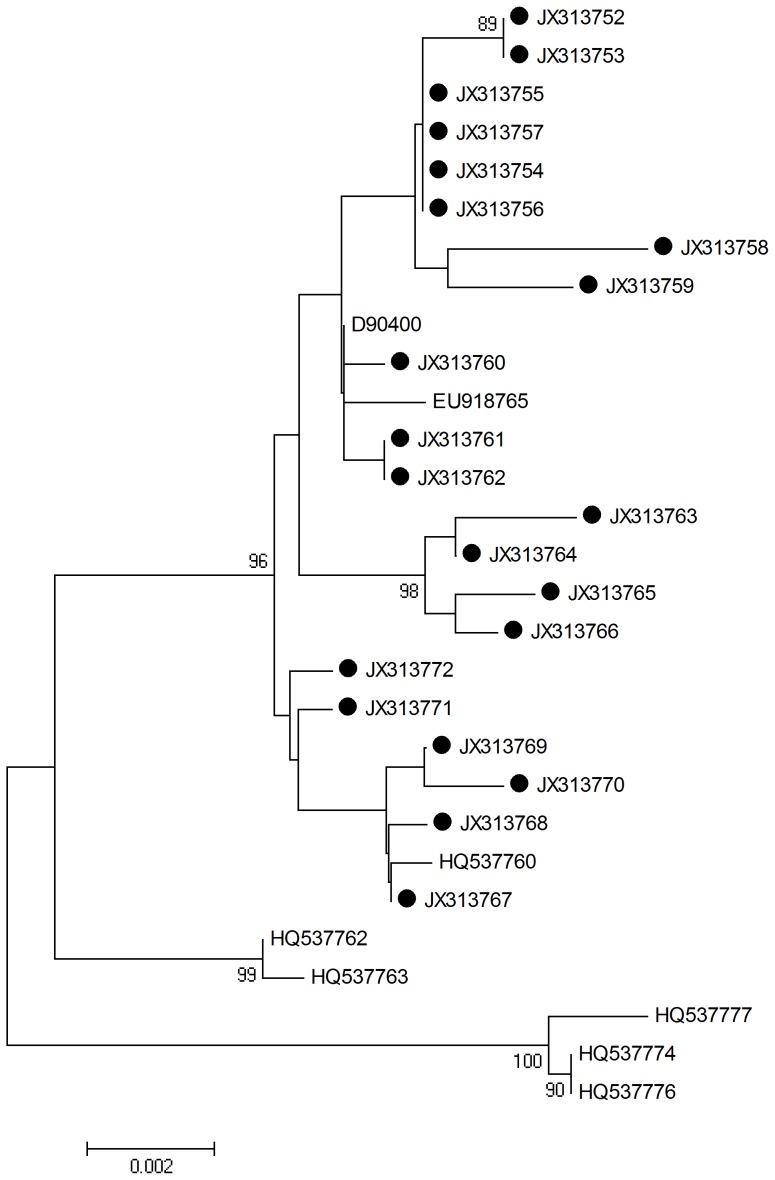
Neighbor joining phylogenetic tree generated using nucleotide sequences of the HPV-58 L1 gene. Study sequences are labeled in black. Others are standard sequences, including: D90400 (Japan), EU918765, HQ537760 (isolate AS347), HQ537762, HQ537763, HQ537776, HQ537774, and HQ537777. Phylogenetic trees were constructed by the Neighbor-Joining method and the Kimura 2-Parameter model by MEGA package.

L1 HPV-58 sequences were determined and analyzed by aligning L1 1575 nucleotide sequences from all viral strains (n = 21; including the reference sequences). The neighbor joining phylogenetic tree can be seen in Fig. 3.

Twenty-four single nucleotide changes were identified among the sequences studied. Specifically, 14/24 (58.3%) were synonymous mutations and 10/24 (41.7%) were non synonymous mutations. 8 amino acid mutations (four non-synonymous) occurred in the sequences encoding the alpha helix, and 4 mutations were observed in the sequences encoding the beta turn with 2 being non-synonymous. 8 samples were found to have the same mutation from C to A at position 1124. The detected mutations are summarized in Table 4.

**Table 4:** Nucleotide sequence mutations of HPV-58 L1.

	Domain: HPV-58 L1 sequence																							
																	1	1	1	1	1	1	1	1
	2	2	2	3	7	8	8	8	8	8	8	8	8	9	9	9	0	0	1	2	2	3	4	1
	4	6	7	0	7	2	2	3	5	5	6	7	7	3	5	9	2	7	2	3	9	1	3	4
	5	4	0	9	5	6	7	0	1	2	2	0	7	6	7	6	9	7	4	3	6	7	7	2
HQ537759	A	A	C	T	G	C	T	G	G	A	A	T	C	C	A	A	A	G	C	G	A	A	A	A
JX313752	.	.	.	.	.	.	.	.	.	.	.	.	.	.	.	G	.	.	A	A	.	.	C	.
JX313753	.	.	.	.	.	.	.	.	.	.	.	.	.	.	.	G	.	.	A	A	.	.	C	.
JX313754	.	.	.	.	.	.	.	.	.	.	.	.	.	.	.	G	.	.	A	.	.	.	.	.
JX313755	.	.	.	.	.	.	.	.	.	.	.	.	.	.	.	G	.	.	A	.	.	.	.	.
JX313756	.	.	.	.	.	.	.	.	.	.	.	.	.	.	.	G	.	.	A	.	.	.	.	.
JX313757	.	.	.	.	.	.	.	.	.	.	.	.	.	.	.	G	.	.	A	.	.	.	.	.
JX313758	.	.	.	.	A	.	.	A	A	.	.	.	.	.	.	G	.	.	A	.	.	.	.	.
JX313759	.	.	.	.	.	T	C	.	A	.	T	.	.	.	.	G	.	.	A	.	.	.	.	.
JX313760	.	.	.	.	.	.	.	.	.	.	.	.	.	.	.	.	.	.	.	.	G	.	.	.
JX313761	.	.	.	.	.	.	.	.	.	.	.	.	A	.	.	.	.	.	.	.	.	.	.	.
JX313762	.	.	.	.	.	.	.	.	.	.	.	.	A	.	.	.	.	.	.	.	.	.	.	.
JX313763	C	.	A	.	.	.	.	.	.	G	.	C	.	.	.	.	.	A	.	.	.	.	.	C
JX313764	.	.	.	.	.	.	.	.	.	G	.	.	.	.	.	.	.	A	.	.	.	.	.	C
JX313765	.	.	.	G	.	.	.	.	.	G	.	C	.	.	G	.	.	A	.	.	.	.	.	.
JX313766	.	G	.	.	.	.	.	.	.	G	.	.	.	.	G	.	.	A	.	.	.	.	.	.
JX313767	.	.	.	.	.	.	.	.	.	.	.	.	.	.	.	.	.	.	.	.	.	.	.	.
JX313768	.	.	.	.	.	.	.	.	.	.	.	.	.	.	.	.	G	.	.	.	.	.	.	.
JX313769	.	.	.	.	.	.	.	.	.	.	.	.	.	T	.	.	.	.	.	.	.	.	.	.
JX313770	.	.	.	.	.	.	.	.	.	.	.	.	.	T	.	.	.	.	.	.	.	G	.	.
JX313771	.	.	.	.	.	.	.	.	.	.	.	.	.	T	.	.	.	.	.	.	.	.	.	.
JX313772	.	.	.	.	.	.	.	.	.	.	.	.	.	.	.	.	G	.	.	.	.	.	.	.
AA mutation	N-T				D-N	L-S(276)	L-S(276)	P-K	R-K		N-Y								T-N				K-T	K-T
second structure	Beta turn				Beta turn			Alpha helix	Alpha helix	Alpha helix	Alpha helix	Beta turn				Alpha helix	Beta turn			Alpha helix		Alpha helix	Alpha helix	

Compared to prototype HPV sequences, neither frame shifts, premature stop codons, insertions nor deletions were observed in the L1 HPV-58 analyzed sequences.

### Phylogenetic and amino acid mutations analysis of HPV-58 L2 sequences

**Figure 4:**
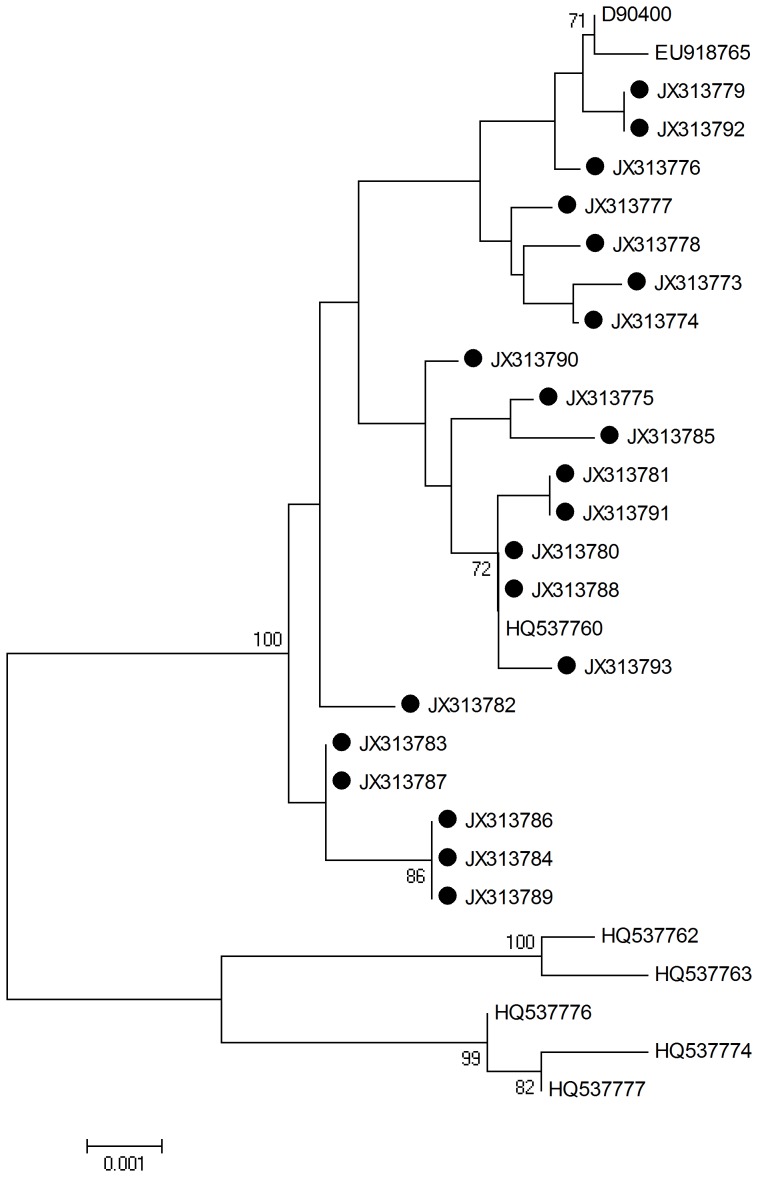
Neighbor joining phylogenetic tree generated using nucleotide sequences of the HPV-58 L2 gene. Study sequences are labeled in black. Others are standard sequences, including: D90400 (Japan), EU918765, HQ537760 (isolate AS347), HQ537762, HQ537763, HQ537776, HQ537774, and HQ537777. Phylogenetic trees were constructed by the Neighbor-Joining method and the Kimura 2-Parameter model by MEGA package.

L2 HPV-58 sequences were determined and analyzed by aligning L2 1419 nucleotide sequences from all viral strains (n = 21; including the reference sequences). The neighbor joining phylogenetic tree can be seen in Fig. 4.

Seven single nucleotide changes were identified among the sequences studied. Specifically, 3/7 (42.9%) were synonymous mutations and 4/7 (57.1%) were non-synonymous mutations. No amino acid changes were observed at residues 65–71 and 112–120, which play an important role in inducing neutralizing antibodies. No amino acid mutations occurred in the sequences encoding the alpha helix and beta turn, but 6 mutations were found in the random coil, and one in the extended strand. The detected mutations are summarized in Table 5.

**Table 5:** Nucleotide sequence mutations of HPV-58 L2.

	Domain: HPV-58 L2 sequence						
	3	6	6	7	7	7	9
	7	1	9	0	2	5	6
	8	6	9	0	7	7	3
HQ537759	A	G	A	C	T	G	A
JX313773	.	.	C	A	.	.	G
JX313774	.	.	C	.	.	.	G
JX313775	.	.	C	A	.	.	.
JX313776	.	.	.	.	.	.	G
JX313777	.	.	.	.	.	.	G
JX313778	.	.	.	.	A	.	G
JX313779	.	.	.	.	.	.	G
JX313780	.	.	.	.	.	.	.
JX313781	.	.	.	.	A	.	.
JX313782	.	.	.	.	.	.	.
JX313783	.	.	.	.	.	.	.
JX313784	C	A	.	.	.	.	.
JX313785	.	.	C	A	.	.	.
JX313786	C	A	.	.	.	.	.
JX313787	.	.	.	.	.	.	.
JX313788	.	.	.	.	.	.	.
JX313789	C	A	.	.	.	.	.
JX313790	.	.	.	.	.	.	.
JX313791	.	.	.	.	A	.	.
JX313792	.	.	.	.	.	.	G
JX313793	.	.	.	.	.	A	.
AA mutation		L-N	Q-H	Q-K		D-N	
second structure	Random coil	Random coil	Random coil	Random coil	Random coil	Random coil	Extended strand.

Insertion and deletion events were not identified and there was no evidence of premature stop codons or nucleotide deletions in the L2 HPV-58 sequences analyzed.

### Selective pressure analysis of all sequences

We tested for variable dN/dS rate ratios among various lineages using the PAML4.0 [25]. There was no evidence of negative selection in the sequence alignment of HPV-16 and HPV-58 L1 and L2 genes (P-value <0.1). The selective pressure analysis results are summarized in Table 6,7,8,9.

**Table 6:** Site-specific tests for positive selection on HPV-16 L1.

Models	lnL	Estimates of parameters	2Δl	Positively selected sites
M7	−2491.42	p = 0.005, q = 0.046		NA
M8	−2479.10	p0 = 0.985, p = 0.005, q = 0.047, p1 = 0.015, ω = 13.30	24.64 P<0.05	35Y**, 207N**, 327T**, 328S*,493K, 503K

**Table 7:** Site-specific tests for positive selection on HPV-16 L2.

Models	lnL	Estimates of parameters	2Δl	Positively selected sites
M7	−2159.05	p = 0.005, q = 0.020		NA
M8	−2150.14	p0 = 0.998, p = 0.009, q = 0.029, p1 = 0.002, ω = 188.75	17.82 P<0.05	330F**, 378F*

**Table 8:** Site-specific tests for positive selection on HPV-58 L1.

Models	lnL	Estimates of parameters	2Δl	Positively selected sites
M7	−2392.27	p = 0.005, q = 0.020		NA
M8	−2389.02	p0 = 0.991, p = 16.456, q = 99.000, p1 = 0.009, ω = 14.706	6.50 P<0.05	5L, 150L*, 276L,

**Table 9:** Site-specific tests for positive selection on HPV-58 L2.

Models	lnL	Estimates of parameters	2Δl	Positively selected sites
M7	−2089.36	p = 0.005, q = 0.047		NA
M8	−2076.71	p0 = 0.982, p = 2.308, q = 99.000, p1 = 0.018, ω = 24.49	25.30 P<0.05	231T*, 233H*, 234K*, 243L*, 446M**

**Table 6** **,** **7** **,** **8** **,** **9:** lnL, the log-likelihood difference between the two models; 2Δl, twice the log-likelihood difference between the two models; The positively selected sites were identified with posterior probability ≥0.9 using Bayes empirical Bayes (BEB) approach. One asterisk indicates posterior probability ≥0.95, and two asterisks indicate posterior probability ≥0.99. NA means not allowed. NS means the sites under positive selection but not reaching the significance level of 0.9.

## Discussion

Human papillomavirus (HPV) vaccines against L1 are now licensed in more than 100 countries. National and regional immunization programs aimed at young adolescent girls have been widely implemented, and include catch-up programs in some countries up to the age of 18 years or older. However these vaccines target only two of the 15 high-risk HPV types, responsible for 70–80% of cervical cancer cases. The prevention of 96% of cervical cancer would require immunity against at least 7 high risk HPV types (HPV-16, 18, 31, 33, 45, 52 and 58) [5]. L2 and other subtypes' L1 are now being used in vaccine research on a broadening scale.

Among 3000 volunteer outpatients investigated in our early study between 2009 and 2011, 646 cases were HPV positive, for a rate of 21.5%. Among the 646 positive samples, 476 cases were of the high risk type (73.7% of the total positive samples), while 170 cases were of the low risk type (26.3% of the total positive samples). The most common HPV high risk subtypes in Southwest China among female reproductive tract infections were HPV-16, 58,18, 31, 33, and 35. The most common low risk subtypes were HPV-6, 11, and 81. Among 476 high risk type positive samples, HPV-16 and 58 were the main high risk subtypes. HPV-16 comprised 217 cases (45.6% of the total high risk subtypes' samples); HPV-58 145 cases (30.5% of the total high risk subtypes' samples). HPV-16 and HPV-58 comprised 362 cases (76.1% of the total high risk subtypes' samples), while all the other high risk subtypes including HPV33,35,18,31,59,66 comprised only 114 cases (24% of the total high risk subtypes' samples) [26]. This may be attributed to the special geographic location where the samples were taken and interactions of different populations in southwest border district of China.

HPV-16 is the most prevalent high-risk types of HPV worldwide, and also the type that is most frequently associated with cancer [27]–[29]. However, the prevalence of HPV-58 and its relative contribution in the development of cervical neoplasia vary greatly in different area worldwide. HPV-58 has previously been reported to be particularly prevalent in some areas of northeastern Asian (China, Korean, Japan), some regions of central and south America, with a significant trend to increased prevalence in line with the increasing severity of lesions [30]–[38]. These data differed from those of the international study reported by Bosch et al., which did not include Chinese women [27]. HPV-58 is rarely detected in the Americas, Europe and Africa, Worldwide, HPV-58 has been found in only 2% of cervical cancers [27]. In contrast, Chan et al. [39] reported that one-third of the women with cervical cancers in Hong Kong were positive for HPV-58, and similarly high rates have also been reported for Chinese populations living in Shanghai (East of China) (30.4% in cervical cancers subgroup) [40], Jiangxi (middle of China) (18.4% in cervical cancers subgroup) [40], and Taiwan (21.0% in cervical cancers subgroup) [41]. Studies from different group suggest that HPV-58 may play a more prominent role in the development of CC in Asia than HPV types 31, 33, and 45, which are more common on other continents [31], [33], [40], [42], [43]. HPV-58 variants carrying E7 C632T (T20I) and G760A (G63S) substitutions may be associated with an increased risk for cervical cancer [39], [43].

Based on previous research of other research group and us, we chose HPV-16 (L1: n = 31, L2: n = 28) and HPV-58 (L1: n = 21, L2: n = 21) samples to explore the intratype variations, construct their phylogenetic trees and estimate selection pressures acting on the L1 and L2 genes in our current study.

It is reported that specific intratype HPV genome variations may be related to virus infectivity, pathogenicity, progression to cervical cancer, viral particle assembly and host immune response [44], [45]. However, there is still no data demonstrating if immunity to one HPV variant can protect against infection from another variant. Thus, identification of HPV genetic diversity in specific clinical settings may prove important for the rational design of diagnostic, therapeutic, and vaccine strategies [46], [47]. The main purpose of our study was to explore the nucleotide variability and phylogeny of high-risk HPV-16 and HPV-58 samples from Southwest China. To the best of our knowledge, this is the first study examining the L1 and L2 proteins of HPV-16 and -58 variants in Southwest China that have considered the complete sequences of the L1 and L2 genes. Information regarding L1 and L2 gene variations of HPV-16 and HPV-58 in the present study could have important implications for diagnosis and formulation of recommendations for the use of second-generation polyvalent HPV vaccines in China.

The neighbor joining phylogenetic tree results showed that the L1 and L2 of HPV-16 and HPV-58 are distributed in two or three standard branches, not in one specific branch. Most HPV-58 L1 and L2 sequences fell into D90400 (Japan), EU918765, HQ537760 (isolate AS347), which belong to the Asian and the European branches. Some mutations occurred in the sequences encoding the alpha helix and beta turn, which influence protein secondary structure. The function of these mutations still needs further investigations. In this study, the most common mutation of HPV-16 L1 was A979C (T327P). This position located in a major common B cell epitope peptide in both mice and humans, which might affect the immunogenicity of HPV-16 L1 [48].

From the result of selective pressure analysis we conclude that most mutations of HPV-16 and HPV-58 L1 and L2 were of positive selection, which indicated that these amino acid changes were beneficial to accommodate the human papillomavirus to its environment.

The L1 and L2 of HPV-16 and HPV-58 had a low rate of nucleotide changes, something that could be attributed to the fact that HPV uses the host cell's DNA replication machinery, which is characterized by proofreading capacity and post replication repair mechanisms [17]. Moreover, many core functions of viral proteins are very important in the viral life cycle, and this may result in selection that restricts the actual number of possible evolutionary events. Some samples did not belong to any standard type branch because of distinctive mutations or the narrow scope on choosing standard types, requiring further efforts of analysis in the future.

Nucleotide substitutions in viral genomes may affect virus assembly, carcinogenic potential, and host immunologic responses [49]. HPV-16 and HPV-58 gene diversity may help us understand the oncogenic potential of these viral strains and how polymorphisms can affect the host response following infection or vaccination.
